# Comparison of Prevalence and Risk Factors of PTSS Between Chinese Patients With Depression and Non-depressed Controls During COVID-19 Outbreak

**DOI:** 10.3389/fpsyt.2021.719931

**Published:** 2022-01-03

**Authors:** Min Peng, Xinran Song, Luyu Liu, Weifeng Zhao, Pingmei Lai, Guanglin Bao, Tianyou Guo, Xiangyang Zhang

**Affiliations:** ^1^Department of Psychiatry, Huazhong University of Science and Technology Union Shenzhen Hospital, Shenzhen, China; ^2^School of Psychology Shenzhen University, Shenzhen, China; ^3^CAS Key Laboratory of Mental Health, Institute of Psychology, Chinese Academy of Sciences, Beijing, China; ^4^Department of Psychology, University of Chinese Academy of Sciences, Beijing, China

**Keywords:** post-traumatic stress disorder, PTSD, posttraumatic stress symptom, PTSS, depression, COVID-19, prevalence, correlates

## Abstract

**Background:** COVID-19 pandemic is a traumatic event all over the world, and may lead to post-traumatic stress symptom (PTSS) in different population who are under the threat of novel corona virus. Therefore, the aim of our study was to compare the prevalence and risk factors of PTSS between Chinese patients with depression and non-depressed controls during the COVID-19 outbreak.

**Methods:** 437 depressed patients and 2,940 non-depressed controls were enrolled in this cross-sectional study between February 14 and May 9, 2020.The Impact of Events Scale-Revised (IES-R), Zung Self-Rating Depression Scale (SDS), Zung Self-Rating Anxiety Scale (SAS) and Pittsburgh Sleep Quality Index (PSQI) were used to evaluate the psychological status of all the participants.

**Results:** The prevalence of PTSS (IES-R ≥ 33) in depressed patients (45.08%) was higher than that in non-depressed controls (5.31%). Patients with depression were 16 times more likely to suffer from PTSS than those without depression. Correlation analyses showed that the IES-R total score was positively correlated with SDS, SAS, and PSQI scores in both depressed and non-depressed groups (Bonferroni corrected all *p* < 0.001). Multiple linear regression analysis showed that SAS score, and PSQI score were independently associated with IES-R total score in both depression and non-depression groups. In depressed patients, education level and duration of media exposure to COVID-19 were positively associated with PTSS, while in the non-depressed group, subjects who were married, in the 31–50 year group or with higher SDS score were more likely to develop PTSS.

**Conclusions:** These results indicate that the prevalence rate of PTSS in patients with depression is very higher than that in subjects without depression. PTSS are associated with a number of socio-demographic and clinical variables.

## Introduction

The COVID-19 pandemic is a global traumatic events (1), which has caused more than 248 million cases, and resulted in more than 5 million deaths worldwide. Nevertheless, the COVID-19 pandemic has also led to a “mental health tsunami”, including depression (2–5), anxiety (6–12), PTSS (13–17), and poor sleep quality (18–23). PTSD (or PTSS) is one of the most common mental distress among this tsunami. Numerous studies have shown that the prevalence of PTSS was high in different populations during the COVID-19 pandemic. For example, meta-analyses have estimated a combined prevalence of 6.5–42.8% for COVID-19 survivors (24), 18% (95% CI: 13–24%) for health care workers, and 12% (95% CI: 8–16%) for the general population (25). Moreover, PTSS has profound interactions with other psychiatric distress, such as depression, anxiety, and sleep quality (26, 27). For example, sleep quality is found to be a predictor variable of PTSS (28), which moderates the relationship between PTSS and anxiety (29).

The relationship between traumatic events, MDD and PTSD is complex (30). Traumatic events are a common risk factor for MDD and PTSD. MDD patients are at greater risk of PTSD than those without depression when they experience a traumatic event (31, 32). MDD patients with co-morbid PTSD tend to have a poorer prognosis (32). PTSD is a common but easily overlooked comorbidity among MDD patients in the face of global traumatic events of COVID-19. Many recent studies have focused on PTSS or PTSD during COVID-19, but these previous studies have mainly focused on COVID-19 survivors (9–11), hospital staff (3, 12–27), teachers (28), and the general population (29–33). However, few researchers have focused on PTSS co-morbidity in MDD patients during the COVID-19 pandemic (34).

Thus, although previous studies have found a high co-morbidity rate between MDD and PTSD (32), the co-morbidity rate of PTSS in MDD patients during COVID-19 and the clinical characteristics of this population are far from being fully explored. Therefore, the objectives of this study were: (1) to explore the co-morbidity rate, clinical predictors, and correlates of PTSS in MDD patients in Shenzhen, China; and (2) to identify sociodemographic or clinical factors associated with PTSS in MDD patients. In this study, we sought to identify independent risk factors for PTSS, further investigate the relationship between MDD and PTSS, develop appropriate management policies and implement practical interventions to improve PTSS in patients with depression during COVID-19.

## Materials and Methods

### Participates

Four hundred and thirty seven depressed patients and 2,940 non-depressed controls were enrolled in this cross-sectional study between February 14 and May 9, 2020.Our epidemiological research teams conducted the data collection. Each team included one doctor, one nurse and one data collector. Prior to the start of the study, dedicated data collectors were trained on the data collection to ensure proper data collection.

In the non-depressed control group, we used a subgroup sampling method. During the initial phase of the pandemic, prevention and control of the COVID-19 outbreak was complicated by the Chinese New Year travel rush. Individuals potentially exposed to COVID-19 virus were quarantined in Nanshan District, Shenzhen for 14 days. The Nanshan District government used epidemiological surveys to trace the chain and network of transmission of COVID-19. The epidemiological investigation was conducted by our 30 epidemiological investigation teams. Our epidemiological survey team invited all individuals who were isolated but not definitively diagnosed with COVID-19 infection to participate in this cross-sectional study in a face-to-face epidemiological survey. In the non-depressed group, we invited 3,775 subjects to participate in the survey. Four hundred and thirty seven children under the age of 18 were excluded because the sample mainly focused on those over 18 years of age. Twenty one individuals over 70 years of age were excluded because of underlying cognitive impairment. In addition, 132 subjects were excluded because they refused to participate or submitted contradictory or careless answers on the SAS or SDS. This left 3,135 subjects afterwards. Of these, 195 had SDS scores ≥50 and were excluded due to an undetermined MDD diagnosis. Finally, a total of 2,940 subjects without depressive symptoms were recruited as non-depressed controls.

In the depressed group, we recruited 437 patients with depression in the outpatient psychiatric clinics of Shenzhen Nanshan People's Hospital. Patients with depression were recruited in two ways. In the first way, psychiatrists randomly recruited 319 depressed patients during routine face-to-face consultations with our first-time patients and follow-up review patients at the psychiatric outpatient clinic of Huazhong University of Science and Technology Union Shenzhen Hospital. In the second way, the psychiatrists invited 118 depressed patients on follow-up visits to participate through the 160 web-based follow-up platform.

The inclusion criteria of patients with depression were as follows: (1) age between 18 and 70 years old; (2) SDS total score ≥ 50; (3) meeting the diagnostic criteria for Major Depressive Disorder (MDD), evaluated by psychiatrists based on ICD-10 criteria; (4) no history of serious physical diseases, such as vital organ failure, severe brain injury and severe cognitive impairment; (5) agreed to participate in this study. The inclusion criteria of non-depressed controls were as follows:(1) age between 18 and 70 years old, (2) SDS score< 50; (3) no history of serious physical diseases;(4) agreed to participate in this study.

This study was approved by the Ethics Review Committee of Huazhong University of Science and Technology Union Shenzhen Hospital. All subjects signed an informed consent form to participate in this study.

### Procedures

All data were collected using detailed standardized self-report questionnaires containing information on sociodemographics (e.g., age, sex, marital status, education level, occupation, history of alcohol and tobacco use, medical history, duration of media exposure to COVID-19) and mental health status. Standardized questionnaires were included in the hospital's psychological assessment system, the 160 web-based follow-up platform, and WeChat. The psychological evaluation system of the hospital was a computer-based psychological assessment system consisting of a number of psychological test scales that allowed an objective assessment of different psychological parameters. Three hundred and nineteen depressed patients were invited face-to-face by psychiatrists and filled out detailed questionnaires through our psychological assessment system. One hundred and eighteen depressed patients were invited and filled out detailed questionnaires through the 160 web-based follow-up platform. In the epidemiological survey, all our non-depressed controls were invited face-to-face. Two thousand nine hundred and forty non-depressed controls completed the questionnaire *via* WeChat (a social media application widely used in China) to reduce the likelihood of exposure to contaminated items. The survey was conducted at the psychiatric outpatient clinic of Huazhong University of Science and Technology Union Shenzhen Hospital, at the participants' homes or hotels.

### Clinical Measurements

#### Depressive Disorder

The Chinese version of the 20-item Zung Self-Rating Depression Scale (SDS) was adopted to access depressive symptoms. The questionnaire consists of 20 items, and each item is scored according to the Likert scale of 4 points, ranging from 1 to 4 points. According to the standard score, a cut-off value of ≥ 50 was used to identify depressive symptoms (33). This SDS cut-off score (cut-off point raw score ≥ 40, index score ≥ 50) was clinically validated with good diagnostic accuracy (sensitivity, 75%; specificity, 75.1%) (34).

#### Post-traumatic Stress Symptom

Post-traumatic stress symptoms were evaluated using the Impact of Events Scale-Revised (Chinese version) (CIES-R). The questionnaire consists of 22 items, each of which is scored according to the Likert scale of 5 points, ranging from 0 to 4 points, with a total score of 0–88 (35). The IES-R contains three subscales: hyperarousal, intrusion and avoidance. The internal consistency of the CIES-R was α = 0.83–0.89 (36).

The IES-R was also categorized as normal (0–23), low levels of PTSS [(24–32); PTSD clinical concern], moderate levels [(33–36); possible diagnosis of PTSD], and extreme levels (>37) (37). A cutoff score of 33 yielded the best values in terms of diagnostic accuracy of PTSD (sensitivity, 91%; specificity, 82%) (38). In this study, the cut-off score for PTSS was based on a total score of 33 on the IES-R (39, 40).

#### Anxiety

The Chinese version of the Zung Self-Rating Anxiety Scale (SAS) was applied to evaluate anxiety related symptoms. This is a scale composed of 20 items, each of which is scored according to the Likert scale of 4 points, ranging from 1 to 4 (41).

#### Sleep Quality

Sleep disturbance was evaluated with the Pittsburgh sleep quality (PSQI). This scale consists of 7 sub-scales and 19 items, and each item is scored on the Likert scale of 4 points, ranging from 0 to 3 points. The 7 sub-scale scores were added up to provide a total PSQI score (42, 43).

#### Media Exposure to COVID-19

The media exposure to COVID-19 information was evaluated by asking participates how long (≤2 h/day, 2–3 h/day, 3–4 h/day, ≥4 h/day) they spent reading or searching information (hour per day) about COVID-19 within 1 week. According to a previous study, subjects exposed to COVID-19 media for ≥4 h per day had significantly higher levels of anxiety than those who used it for ≤2 h (44). Another study showed that students exposed to COVID-19 media for ≥3 h per day had 2.13 times more acute stress symptoms than students exposed to media for <1 h per day (45). Therefore, we chose ≤2 h, 2–3 h, 3–4 h, and ≥4 h as cutoffs.

### Statistical Analyses

Comparisons of clinical and demographic data were conducted using χ2 analysis for categorical data and ANOVA, MANOVA for continuous data. In order to compare the prevalence of PTSS in patients with depression and non-depressed controls, χ2 test and logistic regression were used. In addition, in depression group and non-depressed control group separately, MANOVA and MANCOVA were performed to compare the differences in clinical parameters between subgroups with and without PTSS. Z-test was used for subgroup comparison. Further, Pearson correlation coefficient was calculated to evaluate the correlation between variables. Bonferroni corrections were used to adjust the p-value for multiple comparisons and correlations (α = 0.05/14 = 0.004). At last, we applied multiple regression analyses to investigate the risk factors associated with IES-R total score, including sex, age, education levels, marital status, drinking history, smoking history, SDS score, SAS score, and PSQI score. SPSS software (version 26.0) was used for statistical analysis, setting a 2-tailed *p* = 0.05 as the significant level.

## Results

### Socio-Demographic and Clinical Characteristics

Table 1 compares the socio-demographic and main clinical data between depressed subjects and non-depressed controls (see Table 1 for Chi-square test and MANOVA results). Compared with the non-depressed controls, the depressed patients had younger age, more women, more drinkers, and more were in non-marital status, along with higher SDS score, SAS score, PSQI score, IES-R total score and three subscale scores (all *P* < 0.001). After we controlled for sex, age, education and marital status, MANCOVA results showed that the differences remain significant for SDS score (*F*_1, 3371_ = 6,614.23, *p* < 0.001), SAS score (*F*_1,3371_ = 3,357.76, *p* < 0.001) and PSQI score (*F*_1,3371_ = 1,900.06, *p* < 0.001), IES-R total score (*F*_1,3371_ = 814.44, *p* < 0.001), IES-R Avoidance subscale score (*F*_1,3371_ = 305.62, *p* < 0.001), IES-R Intrusion subscale score(*F*_1,3371_ = 640.08, *p* < 0.001), IES-R Hyperarousal subscale score (*F*_1,3371_ = 1424.18, *p* < 0.001), between the two groups.

**Table 1:** Social demography and clinical characteristics of subjects with depression and non-depressed (x^2^and MANOVA).

	**Depressed group** **(***n*** = 437)**		**Non-depressed group** **(***n*** = 2,940)**			
	**n**	**%**	**n**	**%**	**x^2^**	**p**
*Age*					69.13	<0.001^**^
18–30	235	53.78	980	33.33		
31–50	174	39.82	1,669	56.77		
51–70	28	6.41	291	9.90		
*Sex*					35.68	<0.001^**^
Male	185	42.33	1,692	57.55		
Female	252	57.67	1,248	42.45		
*Education*					6.98	0.07
≤ 9 years	63	14.42	354	12.04		
9–12 years	67	15.33	587	19.97		
12–15 years	96	21.97	674	22.93		
≥15 years	211	48.28	1,325	45.07		
*Marital status*					64.62	<0.001^**^
Single/Divorced/Losing spouse	241	55.15	1,034	35.17		
Married	196	44.85	1,906	64.83		
*Smoking status*					0.85	0.36
Smoker	94	21.51	691	23.50		
Non-smoker	343	78.49	2,249	76.50		
*Drinking status*					12.98	<0.001^**^
Drinker	78	17.85	345	11.73		
Non-drinker	359	82.15	2,595	88.27		
*Media exposure to COVID-19*					6.64	0.08
≤2 h	95	21.74	651	22.14		
2–3h	67	15.33	501	17.04		
3–4h	66	15.11	554	18.84		
≥4 h	209	47.83	1,234	41.97		
	**Mean**	**SD**	**Mean**	**SD**	**F**	**p**
*Age*	32.59	10.25	36.11	10.05	46.38	<0.001^**^
*Clinical assessments*						
SDS total score	63.19	10.92	33.32	6.25	6,873.13	<0.001^**^
SAS total score	51.57	10.38	32.86	5.20	3,557.31	<0.001^**^
PSQI total score	9.49	4.57	3.23	2.35	1,984.97	<0.001^**^
*IES-R*						
Avoidance	10.17	6.75	5.58	4.95	294.55	<0.001^**^
Intrusion	11.18	6.81	5.23	4.12	647.27	<0.001^**^
Hyperarousal	10.12	5.75	3.39	2.91	1,483.32	<0.001^**^
Total score	31.47	17.94	14.21	10.50	824.14	<0.001^**^

^*^p < 0.05,

** *p < 0.01*.

*SD, standard deviation; SDS, Zung's Self-Rating Depression Scale; SAS, Zung's self-rating anxiety scale*.

*PSQI, Pittsburgh Sleep Quality Index; IES-R, Impact of Events Scale-Revised*.

### Comparison of PTSS Prevalence in Depressed Patients and Non-depressed Controls During COVID-19

The prevalence of PTSS (IES-R ≥ 33, possible diagnosis of PTSD) was significantly higher in the depressed group (45.08%, 95%CI: 40.4–49.8%, 197/437) than in the non-depressed control group (5.31%, 95%CI: 4.5–6.1%, 156/2,940) (x^2^ = 642.99, *P* < 0.001).

Moreover, logistic regression analysis showed that the depressed group was 16 times more likely to have PTSS than the non-depressed group (IES-R ≥ 33) (x^2^ = 457.79, *P* < 0.001; odds ratio = 16.00; 95%CI: 12.25–20.89).

In addition, the IES-R total score in the depression group (31.47 ± 17.94) was significantly higher than that in the non-depressed control group (14.21 ± 10.50) (x^2^ = 824.14, *p* < 0.001). We further analyzed data of the depressed and non-depressed groups respectively, using occupation as a subgroup variable. The results showed no significant differences on IRS-R total scores and three subscores between different occupational groups (including peasants, workers, teachers, health care workers, staffs, self-employed, military personnels, freelancers, domestic workers, other workers and unemployed).

### Comparison of Demographic, Clinical Parameters and Risk Factors of PTSS Between Chinese Depressed Patients and Non-depressed Controls

#### Comparison of Demographic, Clinical Parameters and Risk Factors for PTSS in Chinese Patients With Depression

Among patients with depression, compared with the non-PTSS subgroup, the PTSS subgroup had higher education, more time exposure to COVID-19 media coverage and higher SDS score, IES-R total and all three subscores, SAS score, PSQI score (all *P* < 0.05) (see Chi-square test and MANOVA results in Table 2). After adjusting for covariates including age, gender, education, and marital status, there were no significant differences in SDS scores (*F*_1,431_ = 40.18, *P* < 0. 001, Bonferroni corrected), SAS scores (*F*_1,431_ = 91.82, *P* < 0.001, Bonferroni corrected), PSQI scores (*F*_1,431_ = 56.36, *P* < 0.001, Bonferroni-corrected), IES-R total score (*F*_1,431_ = 838.65, *P* < 0.001, Bonferroni-corrected), IES-R avoidance subscale score (*F*_1,431_ = 627.08, *P* < 0.001, Bonferroni-corrected), IES-R interference subscale score (F_1,431_ = 529.99, *P* < 0.001, Bonferroni corrected), and IES-R overanxiety subscale scores (*F*_1,431_ = 551.80, *P* < 0.001, Bonferroni corrected), between the two subgroups (α = 0.05/14 = 0.004).

**Table 2:** Social demography and clinical characteristics of depressed subjects with PTSS and without-PTSS (IES-R ≥ 33) (x^2^ and MANOVA).

	**PTSS group** **(***n*** = 197)**		**Non-PTSS group** **(***n*** = 240)**			
	**n**	**%**	**n**	**%**	**x^**2**^**	**p**
*Age*					2.68	0.26
18–30	104	52.79	131	54.58		
31–50	84	42.64	90	37.50		
51–70	9	4.57	19	7.92		
*Sex*					1.10	0.29
Male	78	39.59	107	44.58		
Female	119	60.41	133	55.42		
*Education*					21.24	<0.001^**^
≤ 9 years	14	7.11	49	20.42		
9–12 years	28	14.21	39	16.25		
12–15 years	40	20.30	56	23.33		
≥15 years	114	57.87	96	40.00		
*Marital status*					0.50	0.48
Single/Divorced/Losing spouse	105	53.30	136	56.67		
Married	92	46.70	104	43.33		
*Smoking status*					2.23	0.14
Smoker	36	18.27	58	24.17		
Non-smoker	161	81.72	182	75.83		
*Drinking status*					2.15	0.14
Drinker	41	20.81	37	15.42		
Non-drinker	156	79.19	203	84.58		
*Media exposure to COVID-19*					8.88	0.03^**^
≤2 h	31	15.74	64	26.66		
2–3 h	33	16.75	39	16.25		
3–4 h	34	17.26	28	11.67		
≥4 h	99	50.23	109	45.42		
	**Mean**	**SD**	**Mean**	**SD**	**F**	**p**
*Age*	32.34	10.07	32.80	10.41	0.22	0.64
*Clinical assessments*						
SDS total score	66.77	11.43	60.26	9.56	42.14	<0.01^**^
SAS total score	56.45	10.34	47.56	8.55	96.87	<0.01^**^
PSQI total score	11.29	3.83	8.02	4.61	63.43	<0.01^**^
*IES-R*						
Avoidance	15.95	4.96	5.43	3.60	657.26	<0.01^**^
Intrusion	16.85	5.25	6.52	3.69	579.48	<0.01^**^
Hyperarousal	14.90	3.66	6.20	3.89	569.86	<0.01^**^
Total score	47.70	11.33	18.16	9.30	895.38	<0.01^**^

*p < 0.05,

** *p < 0.01*.

*SD, standard deviation; SDS, Zung's Self-Rating Depression Scale; SAS, Zung's self-rating anxiety scale*.

*PSQI, Pittsburgh Sleep Quality Index; IES-R, Impact of Events Scale-Revised*.

Correlation analyses revealed that IES-R total score was significantly associated with education levels (*r* = 0.229, *P* < 0.001), media exposure to COVID-19 (*r* = 0.114, *p* = 0.017), SDS score (*r* = 0.418, *P* < 0.001), SAS score (*r* = 0.553, *P* < 0.001), and PSQI score (*r* = 0.457, p < 0.001) (Detailed results of correlation analyses are provided in Table 3). Further, education, SDS score, SAS score, and PSQI score passed the Bonferroni correction (α = 0.05/14 = 0.004).

**Table 3:** Correlations between demographic characteristics and clinical variables of depressed group.

	**Sex**	**Age**	**Marital status**	**Education**	**Smoking**	**Drinking**	**Media exposure to COVID-19**	**SAS**	**SDS**	**PSQI**
Age	−0.103^*^									
Marital status	−0.093	0.567^**^								
Education	0.133^**^	−0.288^**^	−0.239^**^							
Smoking	0.318^**^	0.056	−0.009	0.131^**^						
Drinking	0.217^**^	0.000	−0.012	0.099^*^	0.410^**^					
Media exposure to COVID-19	0.069	−0.282^**^	−0.184^**^	0.131^**^	−0.023	−0.061				
SAS	0.195^**^	−0.115^*^	−0.065	0.132^**^	0.011	−0.114^*^	−0.019			
SDS	0.215^**^	−0.227^**^	−0.152^**^	0.145^**^	−0.034	−0.102^*^	0.044	0.781^**^		
PSQI	0.191^**^	0.069	−0.057	0.181^**^	0.102^*^	−0.057	−0.031	0.534^**^	0.466^**^	
IES-R total	0.066	−0.063	−0.035	0.229^**^	0.083	−0.091	0.114^*^	0.553^**^	0.418^**^	0.457^**^

* *p < 0.05*,

** *p < 0.01*.

*SD, standard deviation; SDS, Zung's Self-Rating Depression Scale; SAS, Zung's self-rating anxiety scale; PSQI, Pittsburgh Sleep Quality Index; IES-R, Impact of Events Scale-Revised; PTSS: post-traumatic stress disorder symptoms*.

Further multiple regression analyses were used to examine the relationship between IES-R scores and other variables in the depression group. The covariates in these stepwise forward input models included those with Ps<0.1 in univariate analyses, including age, gender, education, media exposure to COVID-19, SDS, SAS, and PSQI scores. The results of the multiple linear regression analysis are presented in Table 4. Multiple linear regression analysis showed that education level (β = 0.122, t = 3.121, *p* = 0.002), media exposure COVID-19 (*β* = 0.118, t = 3.068, *p* = 0.002), SAS score (*β* = 0.498, t = 7.759, *P* < 0.001), and PSQI score (*β* = 0.217, t = 4.747, *P* < 0.001) remained associated with the total IES-R score.

**Table 4:** Multiple regression results of IES-R total score and related variables among depressed group.

	**B**	**S.E**	**Beta**	**t**	**P**	**95%CI.for B**	
Education	1.989	0.637	0.122	3.121	0.002^**^	0.736	3.241
Media exposure to COVID-19	1.734	0.565	0.118	3.068	0.002^**^	0.623	2.845
SDS	−0.155	0.101	−0.095	−1.538	0.125	−0.354	0.043
SAS	0.859	0.111	0.498	7.759	0.000^**^	0.642	1.077
PSQI	0.850	0.179	0.217	4.747	0.000^**^	0.498	1.202

*p < 0.05,

** *p < 0.01*.

*SD, standard deviation; SDS, Zung's Self-Rating Depression Scale; SAS, Zung's self-rating anxiety scale; PSQI, Pittsburgh Sleep Quality Index; IES-R, Impact of Events Scale-Revised; PTSS: post-traumatic stress disorder symptoms*.

#### Comparison of Demographic, Clinical Parameters and Risk Factors for PTSS Among Non-depressed Controls

In the non-depressed control group, the PTSS subgroup was older and had higher SDS scores, SAS scores and PSQI scores, and IES-R total and subscale scores compared to the non-PTSS subgroup (all *P* < 0.05) (see Chi-square test and MANOVA results in Table 5). After adjusting for covariates including age, gender, education, and marital status, differences remained significant for SDS scores (*F*_1,2934_ = 107.11, *P* < 0. 001, Bonferroni correction), SAS scores (*F*_1,2934_ = 142.67, *P* < 0.001, Bonferroni correction), PSQI scores (F_1,2934_ = 113.05, *P* < 0.001, Bonferroni corrected), IES-R total score (*F*_1,2934_ = 1,222.74, *P* < 0.001, Bonferroni corrected), IES-R avoidance subscale score (*F*_1,2934_ = 858.85, *P* < 0.001, Bonferroni corrected), and between two subgroups of IES-R intrusion subscale scores (*F*_1,2934_ = 917.70, *P* < 0.001, Bonferroni corrected) and IES-R overanxiety subscale scores (*F*_1,2934_ = 783.55, *P* < 0.001, Bonferroni corrected).

**Table 5:** Social demography and clinical characteristics of non-depressed subjects with PTSS and without-PTSS (IES-R ≥ 33) (x^2^ and MANOVA).

	**PTSS group** **(***n*** = 156)**		**Non-PTSS group** **(***n*** = 2,784)**			
	**n**	**%**	**n**	**%**	**x^**2**^**	**p**
*Age*					6.00	0.05
18–30	38	24.36	942	33.84		
31–50	100	64.10	1,569	56.36		
51–70	18	11.54	273	9.81		
*Sex*					3.22	0.07
Male	79	50.64	1,613	57.94		
Female	77	49.36	1,171	42.06		
*Education*					4.39	0.22
≤ 9 years	27	17.31	327	11.75		
9–12 years	28	17.95	559	20.08		
12–15 years	34	21.79	640	22.99		
≥15 years	67	42.95	1,258	45.19		
*Marital status*					2.89	0.09
Single/Divorced/Losing spouse	45	28.85	989	35.52		
Married	111	71.15	1,795	64.48		
*Smoking status*					1.67	0.20
Smoker	30	19.23	661	23.5		
Non-smoker	126	80.77	2,123	76.5		
*Drinking status*					0.19	0.67
Drinker	20	12.82	325	11.67		
Non-drinker	136	87.18	2,459	88.33		
*Media exposure to COVID-19*					4.53	0.21
≤2 h	24	15.39	627	22.52		
2–3 h	30	19.23	471	16.92		
3–4 h	33	21.15	521	18.71		
≥4 h	69	44.23	1,165	41.85		
	**Mean**	**SD**	**Mean**	**SD**	**F**	**p**
*Age*	38.22	9.46	35.99	10.07	7.33	0.007^**^
*Clinical assessments*						
SDS total score	38.04	7.02	33.05	6.10	97.06	<0.001^**^
SAS total score	37.45	6.87	32.60	4.96	134.59	<0.001^**^
PSQI total score	5.12	3.12	3.13	2.25	109.53	<0.001^**^
*IES-R*						
Avoidance	15.57	3.80	5.03	4.38	869.61	<0.001^**^
Intrusion	13.73	3.23	4.76	3.62	920.21	<0.001^**^
Hyperarousal	9.03	2.33	3.07	2.60	786.86	<0.001^**^
Total score	38.33	4.55	12.86	8.99	1,233.84	<0.001^**^

*p < 0.05,

** *p < 0.01*.

*SD, standard deviation; SDS, Zung's Self-Rating Depression Scale; SAS, Zung's self-rating anxiety scale*.

*PSQI, Pittsburgh Sleep Quality Index; IES-R, Impact of Events Scale-Revised; PTSS: post-traumatic stress disorder symptoms*.

Results of the correlation analyses are summarized in Table 6. Correlation analysis revealed that IES-R total score was significantly associated with sex (r = 0.040, *p* = 0.031), age (r = 0.057, *p* = 0.002), marital status (r = 0.056, *p* = 0.002), media exposure to COVID-19 (r = 0.060, *p* = 0.001), SDS score (r = 0.326, *p* < 0.001), SAS score (r = 0.305 *p* < 0.001), and PSQI score (r = 0.345, *p* < 0.001).Further, age, marital status, media exposure to COVID-19, SDS score, SAS score and PSQI score passed the Bonferroni correction (α = 0.05/14 = 0.004).

**Table 6:** Correlations between demographic characteristics and clinical variables of non-depressed group.

	**Sex**	**Age**	**Marital status**	**Education**	**Smoking**	**Drinking**	**Media exposure to COVID-19**	**SAS**	**SDS**	**PSQI**
Age	−0.046^*^									
Marital status	−0.020	0.531^**^								
Education	−0.015	−0.297^**^	−0.203^**^							
Smoking	0.400^**^	−0.050^**^	−0.027	0.163^**^						
Drinking	0.253^**^	−0.122^**^	−0.063^**^	0.154^**^	0.384^**^					
Media exposure to COVID-19	−0.007	−0.181^**^	−0.143^**^	0.084^**^	−0.036	−0.055^**^				
SAS	0.047^*^	−0.105^**^	−0.074^**^	−0.066^**^	−0.038^*^	−0.043^*^	0.037^*^			
SDS	0.072*^*^	−0.147*^*^	−0.122*^*^	−0.075*^*^	−0.022	−0.027	0.064*^*^	0.587*^*^		
PSQI	−0.004	−0.032	−0.053*^*^	0.042^*^	−0.032	−0.054*^*^	0.101*^*^	0.366*^*^	0.392*^*^	
IES-R total	0.040^*^	0.057*^*^	0.056*^*^	0.005	0.014	−0.041^*^	0.060*^*^	0.305*^*^	0.326*^*^	0.345*^*^

* *p < 0.05*,

** *p < 0.01*.

*SD, standard deviation; SDS, Zung's Self-Rating Depression Scale; SAS, Zung's self-rating anxiety scale; PSQI, Pittsburgh Sleep Quality Index; IES-R, Impact of Events Scale-Revised; PTSS: post-traumatic stress disorder symptoms*.

In addition, we used multiple linear regression analysis to examine the relationship between IES-R scores and other variables in the non-depressed group. The covariates in these stepwise forward input models included those with Ps<0.1 in univariate analyses, including age, gender, marital status, SDS, SAS, and PSQI scores. The results of the multiple linear regression analysis are presented in Table 7. Multiple linear regression showed that age (*β* = 0.072, t = 3.648, *P* < 0.001), marital status (*β* = 0.062, t = 3.125, *p* = 0.002), SDS score (*β* = 0.175, t = 8.203, *P* < 0. 001), SAS score (*β* = 0.127, t = 6.062, *P* < 0.001) and PSQI scores (*β* = 0.235, t = 12.744, *P* < 0.001) remained correlated with total IES-R scores.

**Table 7:** Multiple regression results of IES-R total score and related variables among non-depressed group.

	**B**	**S.E**	**Beta**	**t**	**P**	**95%CI.for B**	
Age	0.076	0.021	0.072	3.648	0.000^**^	0.035	0.116
Sex	0.568	0.355	0.027	1.597	0.110	−0.129	1.265
Marriage	1.354	0.433	0.062	3.125	0.002^**^	0.504	2.204
SDS	0.295	0.036	0.175	8.203	0.000^**^	0.224	0.365
SAS	0.257	0.042	0.127	6.062	0.000^**^	0.174	0.340
PSQI	1.051	0.082	0.238	12.744	0.000^**^	0.889	1.213

*p < 0.05,

** *p < 0.01*.

*SD, standard deviation; SDS, Zung's Self-Rating Depression Scale; SAS, Zung's self-rating anxiety scale; PSQI, Pittsburgh Sleep Quality Index; IES-R, Impact of Events Scale-Revised; PTSS: post-traumatic stress disorder symptoms*.

## Discussion

The main purpose of this research is to explore the co-morbidity rate, clinical predictive factors and correlations of PTSS among MDD patients. There were the following main findings in this study. (1) During the initial stage of COVID-19, there was a high co-morbidity rate of PTSS in MDD patients (45.08%). (2) In patients with depression and non-depressed controls, SDS score, SAS score, and PSQI score were significantly correlated with PTSS. (3) In patients with depression, people with higher education and more time exposure to COVID-19 media coverage were more likely to suffer from PTSS. (4) In non-depressed controls, married subjects and 31~50-year age group were more likely to suffer from PTSS.

### The High Prevalence of PTSS Among Depression

In this study, we found a co-morbidity rate of 45.08% (197/437) for PTSS in MDD patients, much higher than in non-depressed controls (5.31%, 156/2,940). Because few studies have focused on PTSS comorbidity in MDD patients during the COVID-19 pandemic, we compared our results with other populations during the COVID-19 pandemic and with several studies prior to the COVID-19 pandemic. When compared with other populations during the COVID-19 pandemic, we found a significantly higher prevalence of PTSS among MDD patients in our sample (45.08%, 95% CI: 40.4–49.8%) than among adolescent MDD patients in China (34.4%) (46) and among COVID-19 survivors during the COVID-19 pandemic (18.66%, 95% CI: 11.98–25.34%) (47), health care workers (28.0%, 95% CI: 9.5–59.0%) (48) and general population (12.8%) (49). Compared with other studies before the COVID-19 pandemic, our 45.08% prevalence was similar to the co-occurrence of depressive symptoms and PTSS 3 months after the 2016 Salt Lake City tornado (44.94%) (50) and close to the global prevalence estimates of MDD and PTSD in the pre-pandemic Meta-analysis of COVID-19 (52%). In addition, we found that depressed patients were 16 times more likely to have PTSS than non-depressed patients. This result suggests that during the COVID-19 pandemic, patients with MDD were more likely to experience severe psychological distress and PTSS than non-depressed controls and some other high-risk populations.

Despite the high rate of comorbidity of posttraumatic stress symptoms and depression, the underlying mechanisms are far from being fully understood. One hypothesis proposes that the high comorbidity rate may be the result of common susceptibility factors, including biological factors, such as certain brain regions, certain specific genetic polymorphisms, etc. For example, the insular (i.e., insular cortex) has been associated with both PTSD and depression. Furthermore, previous studies have shown genetic correlations of 0.71 to 0.80 between PTSD and depression phenotypes (51). Some specific genetic polymorphisms have been shown to be associated with high rates of combined PTSD and MDD (51). For example, the 5-hydroxytryptamine transporter-linked polymorphic region (5-HTTLPR) has been suggested as a possible candidate gene to regulate emotional responses to traumatic events. the interaction between 5HTTLPR mutations and stressful life events can predict depression and PTSD (46, 47). The A1 allele encoding a type 2 dopaminergic receptor is associated with the comorbidity of PTSD and depression (46). Carriers of the G allele of the OXTR rs53576 gene are more likely to have co-morbid PTSD-depression (48). Defects in the GRK-βarrestin2 machinery would lead to excessive CRF (1) receptor signaling, resulting in PTSD and co morbid PTSD (49). In addition, meta-analysis showed that subjects with specific alleles of the FKBP5 gene were more likely to develop MDD and PTSD (50). Thus, many factors may make depressed subjects more likely to develop PTSD, but further research is needed to explore additional possibilities.

### Common Correlates for PTSS in Both Depressed Patients and Non-depressed Controls

We also found that PTSS level was positively correlated with depression level, which is consistent with previous study showing that initial levels of PTSS predicted depressive symptoms (52).

Despite the SDS score, our study identified several other common correlates for PTSS in both patients with depression and non-depressed controls. We found that the IES-R total score was positively associated with SAS score in both depression and non-depression groups, which was consistent with previous studies on breast cancer survivors (53). Another study on American adolescents also showed that anxiety was the most common psychiatric comorbidities in hospitalized adolescent patients with PTSS (54).

Furthermore, this study illustrated that PTSS were significantly positively correlated with poor sleep quality both in depression and non-depression groups, which was in accordance with previous studies, showing that subjects with better sleep quality reported fewer PTSS during COVID-19. Another study also showed that pre-deployment sleep quality was a predictive feature of post-deployment PTSD in active-duty army (55). Recent studies have shown that the relationship between sleep disorders and PTSD is bidirectional (56). Patients with sleep disturbances prior to a traumatic event are more likely to develop PTSD after exposure to a traumatic event (57). Poor sleep quality is a common symptom and feature of PTSD (56) and a predictor of poor PTSD prognosis (58).

### Correlates of PTSS Specific for Depressed Patients

Recent studies have shown that the frequency of exposure to social media was positively associated with acute stress symptoms and anxiety during COVID-19 (44). For example, Ma et al. found that students exposed to COVID-19 media for ≥3 h per day were 2.13 times more likely to have acute stress symptoms than those exposed to media for <1 h per day (59). Ni et al. reported that subjects exposed to COVID-19 media for ≥4 hours per day had significantly higher levels of anxiety than those who used it for ≤2 hours (44). In the present study, by subgroup comparison, we found that subjects exposed to COVID-19 media ≤2 h per day had a significantly lower incidence of PTSS than those exposed for 3–4 h per day (*P* < 0.05). Furthermore, our multiple linear regression also showed that media exposure to COVID-19 was a predictor of PTSS in the depression group, so we further confirmed that the duration of social media exposure in COVID-19 was also associated with PTSS in depressed patients.

We also found that people with higher education were more vulnerable to PTSS in the depressed group, which is consistent with previous observational studies on Nibel traumatized patients, indicating that subjects with high school education or above are more vulnerable to PTSD (60). However, there was no significant correlation between PTSS and education level in the non-depressed control group.

### Correlates of PTSS Specific for Non-depressed Controls

Further, we found that non-depressed subjects between the ages of 31 and 50 years reported more PTSS. Previous studies have demonstrated that age is an effective risk factor for PTSD. For example, Zhang et al. reported that after the 512 Wenchuan earthquake in China, the 30 40-years-old age group reported more PTSD than other age groups (61). Also, Koirala et al. observed that the 31–45-years-old age group was susceptible to PTSD (60), and Divsalar et al. also observed similar results 12 years after the Bam earthquake in Iran (62). Our results are consistent with those previous results. In addition, our study also found that married respondents were more likely to suffer from PTSS than single respondents in non-depressed respondents, which was in accordance with previous research among war veterans (63).

Some of the limitations of our study need to be addressed. First, given this study was cross-sectional, it cannot infer the causality between PTSS and related risk factors. Further studies of prospective longitudinal designs are needed to confirm the association observed in this study. Second, as all participants were enrolled from Shenzhen, China. The level of exposure to COVID-19 in this region is relatively low (462 cases of COVID-19 as of May 9, 2020). Therefore, we should be cautious in extrapolating these findings to other regions and further studies in other ethnic populations are needed. Third, this is a scale-based study. IES-R was used to quantify PTSS. However, PTSD diagnosis have not been established. The lack of PTSD diagnosis may also limit the effectiveness of this study. Fourth, we had 30 epidemiological study groups, and because the data collected went directly into the electronic database, we could not distinguish whether there were differences in the data collected by the different epidemiological study groups. Fifth, the sample size of this study was moderate, which may limit its generalizability.

In conclusion, our results show that during the COVID-19 outbreak, the prevalence of PTSS in the MDD patients was very high (45.08%), and patients with depression were 16 times more likely to suffer from PTSS that those without depression. Our findings in this study have certain clinical implications, showing that nearly half of MDD patients may have PTSS during the COVID-19 pandemic, even if they did not report a severe traumatic event. Previous studies have indicated that MDD patients with PTSS may have a worse prognosis (32). Therefore, the results of this study remind us that such patients should be identified as early as possible, and we can then better predict the course and prognosis of these patients and further optimize their treatment options.

## Data Availability Statement

The raw data supporting the conclusions of this article will be made available by the authors, without undue reservation.

## Ethics Statement

This study was approved by the Ethics Review Committee of Huazhong University of Science and Technology Union Shenzhen Hospital. The patients/participants provided their written informed consent to participate in this study.

## Author Contributions

MP, XZ, and TG designed the study. MP, XS, LL, WZ, PL, GB, and TG participated in the data collection. MP analyzed the data and drafted the manuscript. XZ revised the manuscript. All authors contributed to and approved the final manuscript.

## Funding

This study was supported by National Natural Science Foundation of China, Grant/Award Number: 31871115.

## Conflict of Interest

The authors declare that the research was conducted in the absence of any commercial or financial relationships that could be construed as a potential conflict of interest.

## Publisher's Note

All claims expressed in this article are solely those of the authors and do not necessarily represent those of their affiliated organizations, or those of the publisher, the editors and the reviewers. Any product that may be evaluated in this article, or claim that may be made by its manufacturer, is not guaranteed or endorsed by the publisher.
